# Ocular Injuries Due to Insect Spines (Ophthalmia Nodosa): Potential Hazard to Motorcyclists

**DOI:** 10.7759/cureus.23084

**Published:** 2022-03-11

**Authors:** Sarah Sathyapriya Tamilarsan, Juanarita Jaafar, Tan Chew-Ean, Nurul Ain Masnon, Wan-Hazabbah Wan Hitam

**Affiliations:** 1 Department of Ophthalmology and Visual Science, School of Medical Sciences, Universiti Sains Malaysia, Kelantan, MYS; 2 Ophthalmology, Hospital Sultanah Bahiyah, Kedah, MYS

**Keywords:** visor, motorbike, insect spine, seta, ophthalmia nodosa

## Abstract

Ocular injury remains a potential hazard to motorcyclists. While the incidence of traumatic penetrating or blunt ocular injury is widely known in the literature, ocular injuries due to insect hair or spine (ophthalmia nodosa) among motorcyclists are scarce or unheard of. Here, we report four cases of ocular injuries caused by insect hair spines among motorcyclists. Patients consist of three males and one female with ages ranging from 18 to 24 years. All patients presented with unilateral ocular irritation after a history of insect entry into the eye while riding a motorcycle. Visual acuity upon presentation ranged from 6/6 to 6/60. Penetration of setae into the cornea and anterior chamber reaction was found in all patients. Complete removal of cornea setae was not possible in all patients. Immediate treatment with topical antibiotics and corticosteroids was administered and continued for one to three months. All patients recovered well attaining a vision of 6/6 to 6/9. In conclusion, ophthalmia nodosa among motorcyclists is a preventable ocular hazard with the appropriate use of a visor or protective eyewear. Immediate treatment may prevent severe ocular complications.

## Introduction

Ophthalmia nodosa is defined as an inflammatory reaction in the eye caused by hairs or spines (setae) of certain insects or vegetable material. Insect setae are known to cause several multiple systemic or local effects such as respiratory difficulties, ocular problems, sore throat, and skin irritation [1]. The ocular implication of insect or vegetative setae is infrequent and mostly self-limiting. However, if left untreated, though rare, it has the potential to cause severe ocular damage in humans. Known sources of ophthalmia nodosa include oak caterpillar, tarantula hair, moth, and butterflies [1]. While it is known as an occupational disease in Eastern Mediterranean countries and a potential hazard for ocular and adnexal injury among children, little is known about the ocular impact among motorcyclists [2]. Motorcyclists who do not use helmets with visors are more likely exposed to dust particles, debris, and insect that may often plunge into the eye at high speed. The force and speed with which these insect or vegetative setae enter the eye lead to deeper penetration of the setae into the eye [3]. Thus, motorcyclists are more vulnerable to the more grave consequences of the usual self-limiting disease as deeper intraocular penetration of setae is anticipated. Here, we report four cases of ophthalmia nodosa affecting motorcyclists, along with its clinical presentations, management, and outcomes.

## Case presentation

Case 1

**Figure 1:**
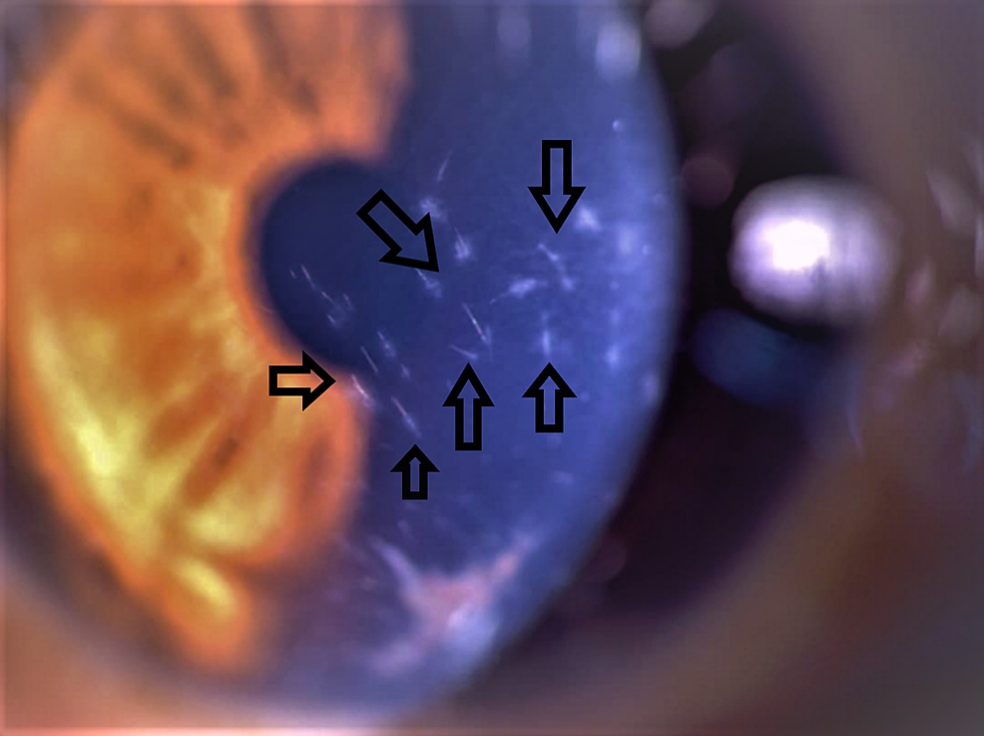
Anterior segment image showing multiple fine setae embedded in the cornea with endothelial pigmentation.

**Figure 2:**
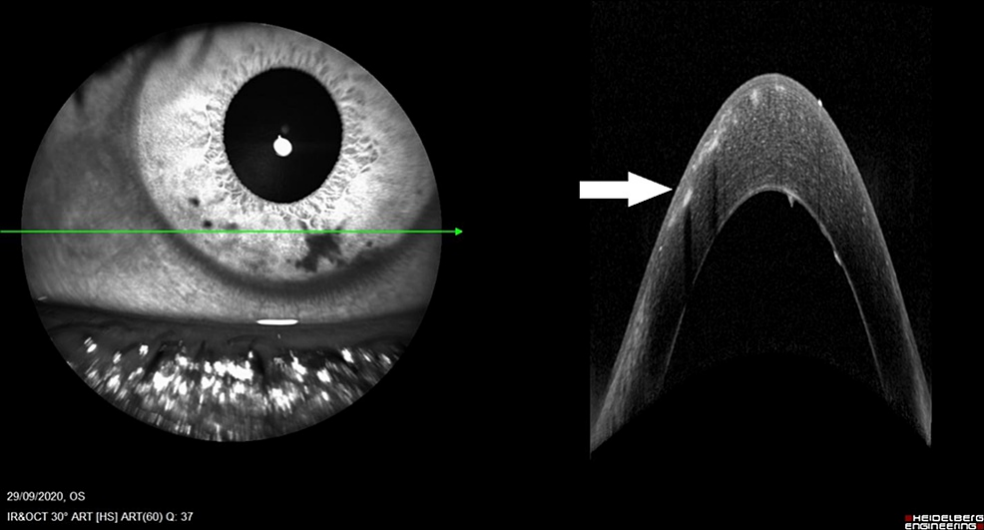
Anterior segment optical coherence tomography image showing evidence of intracorneal setae.

An 18-year-old male presented with left eye redness, photophobia, and epiphora for two weeks. His symptoms were preceded by a history of an unknown insect that collided with his eye while riding his motorcycle. The patient was treated with topical Gutt Chloramphenicol 0.5% every six hours in a private clinic elsewhere; however, his symptoms persisted. On examination, the left visual acuity was 6/60. Approximately 20 setae were embedded in the cornea with surrounding edema (Figure 1). There were two setae in the anterior chamber with the presence of anterior chamber reaction. Some of the setae were removed with microforceps and a 30-gauge needle at the slit lamp over subsequent visits to the clinics whenever the setae resurfaced. Some remained intracorneal and deeply embedded. Anterior segment optical coherence tomography (OCT) showed intracorneal setae (Figure 2). Topical antibiotics, Gutt Vigamox 0.5% and topical corticosteroid, Gutt Maxidex 0.1% were initiated every four hours and gradually tapered over a period of two months. Setae in the anterior chamber disappeared after one month. There was a reduction in the number of setae in the cornea. Gonioscopy showed no evidence of setae in the anterior chamber angles. Corneal edema resolved leaving pigmentation on the endothelium. The patient’s visual acuity improved to 6/9 after two months.

Case 2

**Figure 3:**
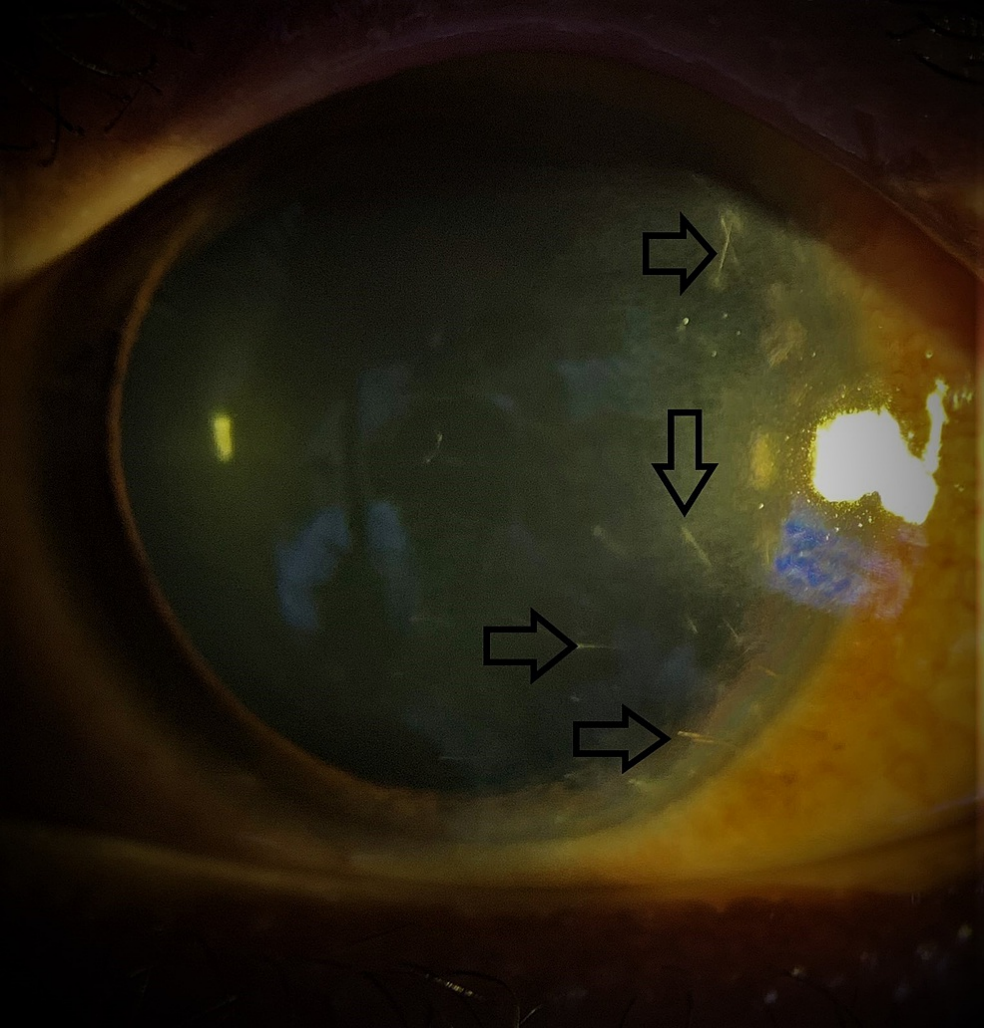
Anterior segment image showing fine setae embedded in the cornea.

**Figure 4:**
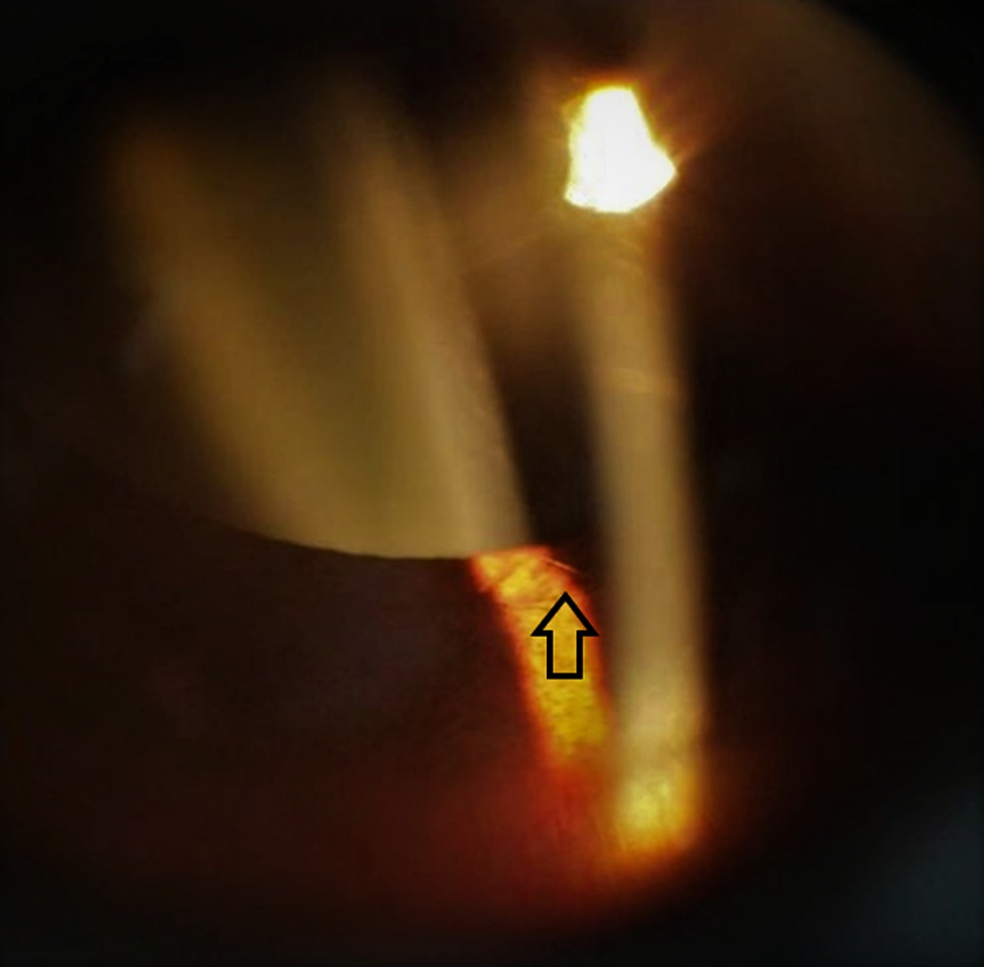
Anterior segment image showing one setae strand that migrated into the anterior chamber.

A 22-year-old man presented with left eye redness, tearing, and itchiness for one week. He was struck by a moth while riding his motorcycle. On examination, the left visual acuity was 6/6 with four setae embedded in the peripheral cornea (Figure 3). The anterior chamber was quiet. Setae could not be removed even with microforceps due to their fine nature. The setae were deeply embedded and protruding into the anterior chamber. Gutt Vigamox 0.5% every 6 hours and Gutt Prednisolone acetate 1% every four hours were initiated and gradually tapered over a period of two months. After one month, there was evidence of migration of setae to the anterior chamber with anterior chamber reaction (Figure 4). Topical medications were gradually tapered for three months until the setae in the cornea and the anterior chamber were completely cleared and quiet. No setae were detected in the anterior chamber angles with gonioscopy.

Case 3

**Figure 5:**
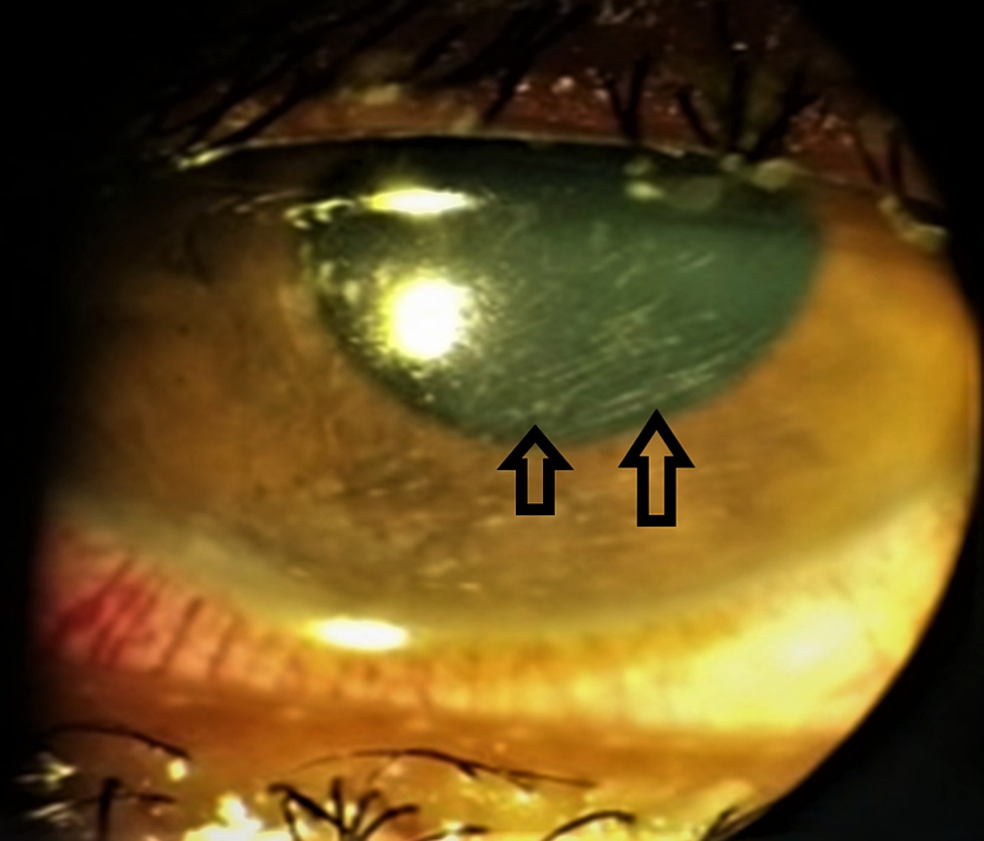
Anterior segment image showing multiple fine setae embedded in the cornea.

A 24-year-old male presented with blurring of vision, redness, and tearing of the right eye for one month. His right eye was hit by an insect while riding his motorcycle along a paddy field. The patient was treated in a private clinic with topical Gutt Olopatadine 0.2%, once daily but with no relief. Upon review, his right visual acuity was 6/36. There were some fine setae that were buried in the upper and lower fornix which was removed with microforceps. Conjunctiva was injected and there were multiple setae embedded in the cornea with severe edema surrounding them. There was anterior chamber reaction despite no setae seen in the anterior chamber or in the angles with gonioscopy. Some protruding setae were removed with a 30-gauge needle at the slit lamp. However, most setae retained intracorneal. Gutt ciprofloxacin 0.3% and Gutt prednisolone acetate 1% were started every four hours. The corneal edema resolved. Setae in the cornea remained in situ (Figure 5). His right eye visual acuity improved to 6/12, and the anterior chamber was quiet after one month.

Case 4

**Figure 6:**
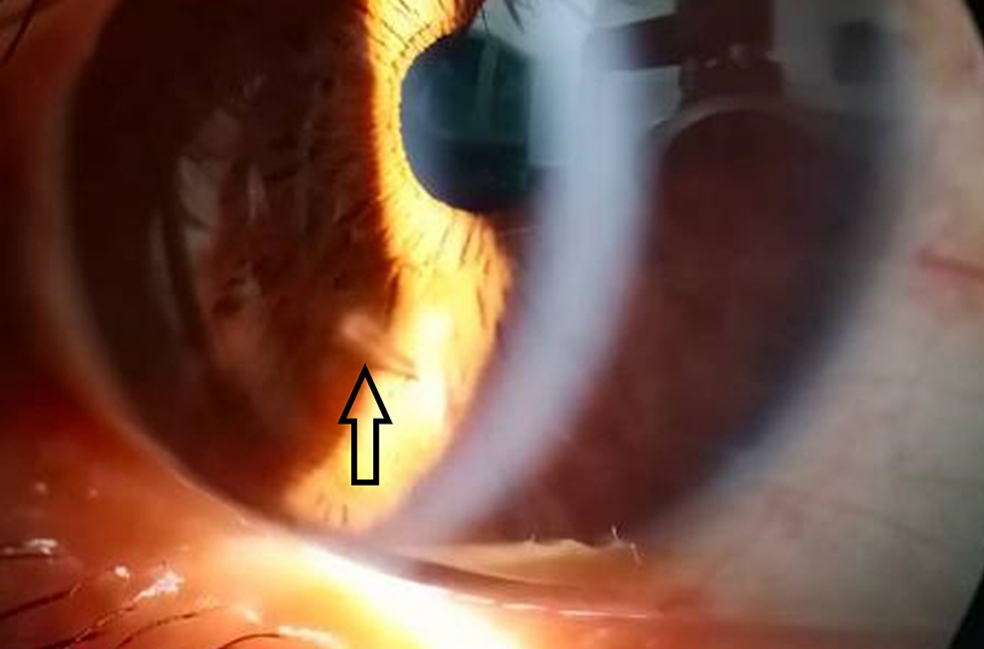
Anterior segment image showing intracorneal setae.

A 23-year-old female presented with left eye discomfort and blurring of vision. She was hit by an insect while riding a motorcycle. Her left visual acuity was 6/18. There was corneal edema with the presence of three setae embedded in the cornea. There was a mild anterior chamber reaction. However, there were no setae in the anterior chamber or in the angles with gonioscopy. Surgical removal of setae with microforceps and corneal toilet and suturing were done. One small seta remained (Figure 6). Gutt Vigamox 0.5% and Gutt Maxidex 0.1% were initiated every four hours. On follow-up after one month, her left eye visual acuity improved to 6/6. Sutures were removed and there was minimum corneal scarring. The anterior chamber was quiet.

**Table 1:** A summary of the cases of ophthalmia nodosa affecting motorbike riders in this case series. AC: anterior chamber; G: Gutt; VA: visual acuity

Case	Age, Sex	Laterality	Visit after onset	VA on presentation	Ocular findings	Treatment	Treatment duration	Final VA	Outcome
1	18, M	LE	Two weeks	6/60	20 setae on the cornea, two setae in AC + AC reaction	Setae removal at the slit lamp. G Vigamox four hourly and G Maxidex four-hourly	Two months	6/9	Intracorneal setae remained, setae in AC completely resolved
2	22, M	LE	One week	6/6	Four setae on the cornea, migrated to AC + AC reaction	G Vigamox six-hourly and G Pred Forte four-hourly	Three months	6/9	Intracorneal and AC setae completely resolved
3	24, M	RE	One month	6/36	15 on the cornea, nil in the AC + AC reaction	Setae removal at slit lamp G Ciprofloxacin four-hourly and G Pred Forte four-hourly	One month	6/12	Intracorneal setae remained
4	23, F	LE	Hours	6/18	Three setae on the cornea, nil in the AC + AC reaction	Surgical removal of setae with corneal toilet and suturing. G Vigamox four-hourly and G Maxidex four-hourly	One month	6/9	Intracorneal setae remained

## Discussion

The occurrence of this condition is rarely reported in the literature. A national survey together with the Dutch Ophthalmic Society found that the oak caterpillar was a significant source of ophthalmological injuries [1]. Interestingly, in Mediterranean countries, ophthalmia nodosa is encountered as an occupational disease as caterpillars are used as a feed for breeding bugs that are used as a biological weapon against caterpillars that destroy red pine trees [2]. Similarly, caterpillars also account for many cases of ophthalmia nodosa in India, as reported in several studies, mostly occurring seasonally from July to October in the coastal region [3,4]. Motorcyclists who do not wear appropriate helmets with visors are often at high risk of injuries as they do not have a protective shield for the eye when insects or airborne setae strike the eye at high speeds.

The pathological damage caused by setae is due to its direct toxicity and locomotion into ocular structures [4]. Setae contain venom gland in their hollow shaft that gets transmitted upon contact with ocular structures. The setae release noxious toxins resulting in granulomatous inflammation in the conjunctiva and episclera, whereas, in the stiffer cornea and sclera, such nodules are not evident [3]. In many cases, these insects setae, especially caterpillars, tend to be sharp and barbed, and due to the direction of the barbs, it usually travels base forward [5]. The postulated theory for intraocular penetration and migration of the setae is related to the movement of the globe with versions, respiration, and iris pulsation as the setae themselves do not have the propulsive ability [6]. Inflammatory exudates pushing over the broken ends of the setae may also contribute to the propulsive effect [7].

Cadera et al. have classified the entire spectrum of ocular pathology caused by caterpillar setae and formulated a treatment guideline [5]. Based on the Cadera classification, most of our patients fall under types 3 and 4. Motorcyclists are more vulnerable to deeper intraocular penetration and migration as the force with which these insect setae strike the eye is usually high [3]. According to Sengupta et al., the risk of migration is higher in the presence of intracorneal hair and the force with which the setae hit the eye [3]. Intraocular penetration of setae can lead to mild-to-severe anterior segment reaction, as observed in all our patients [4,8]. Penetration of setae in the cornea was also accompanied by localized edema around the embedded setae. In cases 1 and 2, ocular inflammation was more prolonged taking about two to three months due to the active migration of setae into the anterior chamber. Whereas in cases 3 and 4, the eye became quiet within one month as the setae were confined intracorneal with no migration. Complete removal of setae may reduce the chances of further migration of setae; however, it becomes a challenge when multiple fine setae lodge in the cornea. Localized nummular keratitis with bouts of recurrence of uveitis and migrating setae was observed in a case report by Ambhasta et al. [6]. The phase of inflammation in the anterior segment is more prominent when the setae are free or protruding in the anterior chamber which is preceded by a quiescent interval during which the setae migrate through ocular structures [9].

The reduction of setae from the cornea and anterior chamber during subsequent follow-ups may likely be due to complete resolution or migration of setae from the anterior segment to posterior segment as it is also known to occur in other reported cases [9]. In two of our patients (cases 1 and 2), setae detected initially in the anterior chamber were no longer visible during the subsequent follow-ups. Possible migration to the posterior chamber was speculated as it can happen without any subsequent vitritis or iridocyclitis, although no visible setae were detected in the posterior segment. This is similar to the case reported by Ibarra et al. [9], whereby intraretinal and corneal setae were noted to be embedded with only minimal and tolerable inflammation without the need for prolonged corticosteroids, laser photocoagulation, or surgery. Surgical intervention to remove seta was not required in any of our cases. Though rare, intraocular setae in the posterior segment that require pars plana vitrectomy with prophylactic intravitreal antibiotics and subconjunctival corticosteroids were reported by Tan et al. [1]. Despite adequate treatment, late-onset endophthalmitis has been noted [3].

The diagnosis of ophthalmia nodosa is often challenging as the variable coloration of setae can result in setae being overlooked during examinations. Anterior segment OCT can be used to detect intracorneal setae, especially if they are not clearly visible, as reported by Taksiran et al. [2]. Persistent unilateral eye redness, especially after possible trauma due to contact with animal setae or vegetative setae, should be examined thoroughly for the presence of setae and considered as a differential diagnosis.

The occurrence of ophthalmia nodosa can be prevented by wearing personal protective equipment and providing proper education to high-risk occupational groups, such as workers in caterpillar-feeding parasite breeding farms and motorcyclists or bicycle riders. Exposure to these offending insects or vegetative materials can further be reduced by wearing protective glasses while working and helmets with visors while riding two-wheelers. The management of ophthalmia nodosa can be divided into primary and secondary care. Primary care is to rinse the eye with clean water thoroughly. Secondary care depends on the type of the disease. Types I to III usually respond well to the removal of setae and standard topical medications, which comprises topical antibiotics and topical steroids, whereas types IV to V may require more invasive interventions. Aggressive treatment for inflammation can be done by starting topical steroids such as topical Gutt Maxidex 0.1% or Gutt Prednisolone acetate 1% every two to four hours. Prophylactic antibiotics from the quinolone group such as G ciprofloxacin 0.3 % or G Vigamox 0.5% every two to four hours are initiated to prevent complicated infections such as keratitis, anterior uveitis, and endophthalmitis. Removal of setae can be attempted with forceps, iridectomy, lensectomy, and Nd:YAG laser to disrupt setae. Pars plana vitrectomy may be required with the presence of setae in the vitreous or consequent endophthalmitis [1].

The prognosis of ophthalmia nodosa is good as it is generally self-limiting. The removal of setae also does not warrant an uneventful recovery as the toxins released by setae and the track it creates in the eyes always leave a pathway for secondary bacterial infections. Hence, there is a need for thorough and repeated examinations on each visit as patients are likely to be more cooperative at subsequent examination once inflammation subsides.

## Conclusions

Ophthalmia nodosa in motorcyclists is a preventable ocular hazard with the appropriate use of visor or protective eyewear. It is usually a self-limiting condition. Topical corticosteroids may prevent chronic inflammation in the anterior segment. Retained intraocular setae may lead to recurrent intraocular inflammation. Immediate treatment may prevent severe ocular complications. Outcomes in most cases are good.
